# Deployment of spatial attention without moving the eyes is boosted by oculomotor adaptation

**DOI:** 10.3389/fnhum.2015.00426

**Published:** 2015-08-04

**Authors:** Ouazna Habchi, Elodie Rey, Romain Mathieu, Christian Urquizar, Alessandro Farnè, Denis Pélisson

**Affiliations:** Integrative, Multisensory, Perception, Action and Cognition Team, Lyon Neuroscience Research Center, INSERM, Unit 1028, CNRS Unit 5292, Bron, France and Lyon I UniversityLyon, France

**Keywords:** exogenous attention, reactive saccade, voluntary saccade, saccadic adaptation, covert attention

## Abstract

Vertebrates developed sophisticated solutions to select environmental visual information, being capable of moving attention without moving the eyes. A large body of behavioral and neuroimaging studies indicate a tight coupling between eye movements and spatial attention. The nature of this link, however, remains highly debated. Here, we demonstrate that deployment of human covert attention, measured in stationary eye conditions, can be boosted across space by changing the size of ocular saccades to a single position via a specific adaptation paradigm. These findings indicate that spatial attention is more widely affected by oculomotor plasticity than previously thought.

## Introduction

Evolution has provided vertebrates with advanced systems allowing attention to be directed elsewhere from where the eyes look (Posner, 1980). Typically, we select visual information via overt movements of the eyes (saccades) and covert shifts of attention (without saccades). Although the functional coupling between eye movements and spatial attention mechanisms is central to prominent theories of attention control (Rizzolatti et al., 1987; Sheliga et al., 1994; Hoffman and Subramaniam, 1995; Kowler et al., 1995; Deubel and Schneider, 1996; Nobre et al., 1997; Beauchamp et al., 2001; Craighero et al., 2004; Corbetta et al., 2008; Smith and Schenk, 2012), the conditions in which this coupling exists remain highly debated. For example, contrasting with the original version of the pre-motor theory of attention, Smith and Schenk (2012) proposed that this coupling exists mainly in the particular case of the pre-saccadic shift of attention and of exogenous attention. A successful tool to qualify this coupling consists in testing visual detection or discrimination abilities—as proxies for attention deployment—after temporary modification of eye movements’ size through saccadic adaptation. Adaptation is a plastic re-calibration of the motor commands that compensates for saccade execution errors (Hopp and Fuchs, 2004; Pélisson et al., 2010; Prsa and Thier, 2011; Herman et al., 2013) and that can be induced non-invasively in the laboratory by using the double-step target paradigm (McLaughlin, 1967). To date, scholars have found that the enhancement of visual perception typically occurring at the landing position of an upcoming saccade—called pre-saccadic shift of attention—follows the new metrics of eye movement induced by adaptation (Doré-mazars and Collins, 2005; Collins and Doré-Mazars, 2006; Collins et al., 2010; Khan et al., 2010). However, the discovery that brain lesion can selectively disrupt the ability to orient covert attention without compromising the pre-saccadic shift of attention (Blangero et al., 2010), casts serious doubts on their supposedly intimate relationship (Smith and Schenk, 2012). Therefore, whether saccadic adaptation can modulate the deployment of pure covert attention remains unknown. In addition, we were also interested to assess whether such modulation is specific to the type of adapted saccades, given the known differences of adaptation properties and neural substrates between reactive saccades (RS), elicited automatically in response to a change in the visual display, and voluntary saccades (VS) elicited when scanning a stationary visual display (Zimmermann and Lappe, 2009; Pélisson et al., 2010; Gerardin et al., 2012; Panouillères et al., 2014). Resolving these issues will provide strong insight into the nature and possible neural substrates of the link between the eye movements and attention systems.

Here, we investigated directly these questions by testing in healthy subjects the effects of saccadic adaptation on spatial attention as indexed by two visual tasks where saccadic eye movements were prohibited. In Experiment I, a simple detection task was performed before and after adapting, in a between subjects design, RS and scanning VS. In Experiment II, a spatial discrimination task was used, in a within subject design, to ascertain the nature of the changes in visuospatial attention following RS adaptation.

## Materials and Methods

### Experiment I

#### Subjects

Seventy-three healthy volunteers were recruited for Experiment I [35 females and 38 males, 71 right-handed and 2 left-handed, mean age: 24.66, Standard Error of Mean (SEM ± 0.69)]. All subjects had a normal or corrected-to-normal vision, and had no history of neurological or psychiatric disorder. All subjects gave their informed consent to participate to the study, which lasted less than 1 h. The experiment conformed to the code of ethics of the World Medical Association – Declaration of Helsinki (2008) and all procedures were approved by the local ethics committee.

Subjects were pseudo-randomly assigned (taking into account age and gender) to one of four groups of the RS condition or one of the 4 groups constituting the VS condition; five of the subjects were enrolled in more than one group (four subjects in two groups and one subject in three groups) and were tested at intervals ranging from 1 to 4 months. Each RS and VS condition followed a 2 × 2 factorial design with the factors “hemi-field” (left vs. right) and “saccade task” (adaptation vs. control).

Two groups (*N* = 10 each) performed a RS adaptation protocol in either the left hemi-field (Adapt-Left: 5 Females, mean age 23.4, SEM ± 3.2) or the right hemi-field (Adapt-Right: 4 Females, mean age 24.7, SEM ± 2.5); two other groups (*N* = 10 each) serving as controls performed a RS control task either in the left hemi-field (Control-Left: 5 Females, mean age 25.5, SEM ± 1.6) or right hemi-field (Control-Right: 6 Females, mean age; 25.9, SEM ± 1.0). Two groups were submitted to a VS adaptation protocol in either the left hemi-field (Adapt-Left: 5 Females, mean age 24.2, SEM ± 1.2) or right hemi-field (Adapt-Right: 5 Females, mean age 24.1, SEM ± 0.64) and two control groups performed a VS control task in either hemi-field (Control-Left: 5 Females, mean age 23.6, SEM ± 0.84; Control-Right: 5 Females, mean age 26.6, SEM ± 1.90).

#### Apparatus

Subjects sat in a dimly lit room, 57 cm away from a 17-inch (30° × 40° of visual angle) computer screen (140 Hz) with their head stabilized by a chin rest, cheekbone rests, and forehead support. Visual stimuli (0.6° diameter black dots on a grey background) were presented using a Visual Stimuli Generation system (Cambridge Research Systems, Cambridge, UK). Binocular eye movements were recorded at a frequency of 500 Hz and spatial resolution of 0.05° using an infrared tracker (EyeLink 1000, SR Research, Canada). A calibration of the Eye tracker was performed before each recording session by asking subjects to serially fixate nine dots constituting a rectangle (28° × 38°) covering the computer screen surface. Laboratory-developed software coupled with a real-time interface allowed on-line monitoring of eye movements and triggering of the visual stimulation. Eye movement data were stored for off-line analysis. Key press responses in the visual detection task were collected using a button box located along the subject’s body midline.

#### Procedure

**Figure 1:**
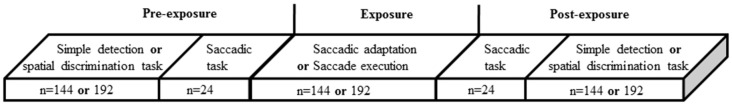
**General flow chart of experiments**. Each subject performed five blocks of trials: in Experiment I, a detection block and a saccade block performed twice (in the pre- and the post-exposure phases), and an adaptation or a control block performed during the exposure phase (in Experiment II detection was replaced by spatial discrimination). The number of trials in each block (n) is indicated.

Each of the four sessions per condition (2 saccade tasks × 2 hemi-fields) involved three phases: pre-exposure, exposure and post-exposure (Figure 1). The exposure phase consisted in either a saccadic adaptation task or its corresponding control task (mere execution of saccades) with visual targets presented in either the left or right hemi-field. Pre-exposure and post-exposure phases were identical, comprising a saccadic task and a visual detection task performed sequentially, each task measuring performance in both hemi-fields.

##### Exposure phase of reactive saccade condition

**Figure 2:**
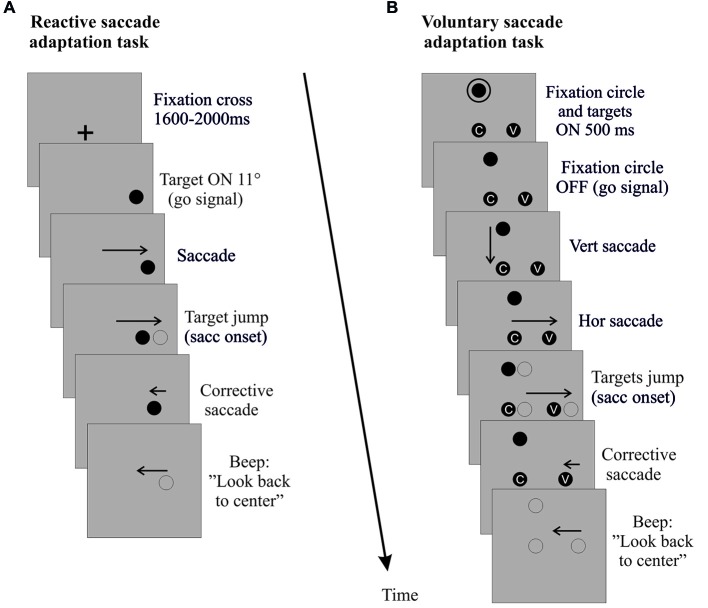
**Saccade adaptation exposure**. Sequences of events (time running downward) in right hemi-field trials (left hemi-field trials not shown) are depicted for the reactive saccade adaptation task **(A)** and the voluntary saccade adaptation task **(B)**. Vertical and rightward horizontal arrows correspond to the primary saccade toward the target. Leftward arrows correspond to the corrective saccade toward the shifted target and to the return saccade to the screen center. When the horizontal primary saccade is detected (eye velocity threshold 70–90°/s), the target (reactive saccade adaptation) or the entire visual display (voluntary saccade adaptation) jumps back toward the center by 4°. For the control tasks (not shown), the same sequence of events unfolds, except for the target jumps.

The classical double-step paradigm (McLaughlin, 1967) was used to induce backward adaptation of RS. This paradigm consists in systematically shifting the target in the direction opposite to, and at the onset of, each horizontal primary saccade. At the beginning of each adaptation trial (Figure 2A), a central fixation cross (FC) was presented. After a random delay (1600–2000 ms) the FC was turned off and simultaneously a target appeared at an eccentricity of 11°, in either the right hemi-field (Adapt-Right group) or left hemi-field (Adapt-Left group). Subjects were instructed to look at the target as soon as it appeared. When the horizontal saccade was detected (eye velocity threshold: 70–90°/s, on average 9 ± 0.6 ms after the saccade onset as measured off-line) the target was shifted backward from 11° to 7° (i.e., a target step corresponding to 36% of the initial target eccentricity). The displaced target remained visible for 500 ms after the end of the horizontal saccade. At the end of each trial, a beep indicated the subjects to look back to the center of the screen and prepare for the next trial. The FC reappeared 1200 ms after the beep. This adaptation exposure phase was composed of three blocks of 48 trials.

In the control task, participants performed RS directed either to the left hemi-field (Control-Left group) or to the right hemi-field (Control-Right group). This control task was identical to the adaptation task, except that the target was presented randomly at 11° or 7° with equal probability and did not jump at saccade onset. The control exposure phase was composed of three blocks of 48 trials.

##### Exposure phase of voluntary saccade condition

A modified double-step paradigm (Panouillères et al., 2014) was used to induce backward adaptation of VS (Figure 2B). At the beginning of each trial, subjects had to look at a fixation point (FP) located 7° above the horizontal meridian. After 1600 ms, a circle appeared around the FP concurrently with two targets: one at 7° below the FP (screen center) and another located at 11° along the screen horizontal meridian either in the right hemi-field (Adapt-Right group) or in the left hemi-field (Adapt-Left group). After 500 ms the circle turned off, signaling subjects to perform first a vertical saccade to the central target, then a horizontal saccade toward the lateral target. To enforce attentive fixation of each target, subjects were instructed to fixate a small grey letter inside the central and lateral targets. When the horizontal VS was detected (eye velocity threshold: 70–90°/s), the FP and the two targets were shifted backward by 4° (i.e., by 36% of the lateral target initial eccentricity). At the end of each trial, a beep indicated the subjects to look back to the center of the screen and to prepare for the next trial. The number of trials and size of intra-saccadic target steps were identical to those in the RS condition.

In the control task, participants performed VS directed either to the left hemi-field (Control-Left group) or to the right hemi-field (Control-Right group). This control task was identical to the adaptation one, except that the lateral target was located at 7° or 11° with equal probability and that no target jumped at saccade onset. The control exposure phase was composed of three blocks of 48 trials.

##### Pre- and post-exposure phases

**Figure 3:**
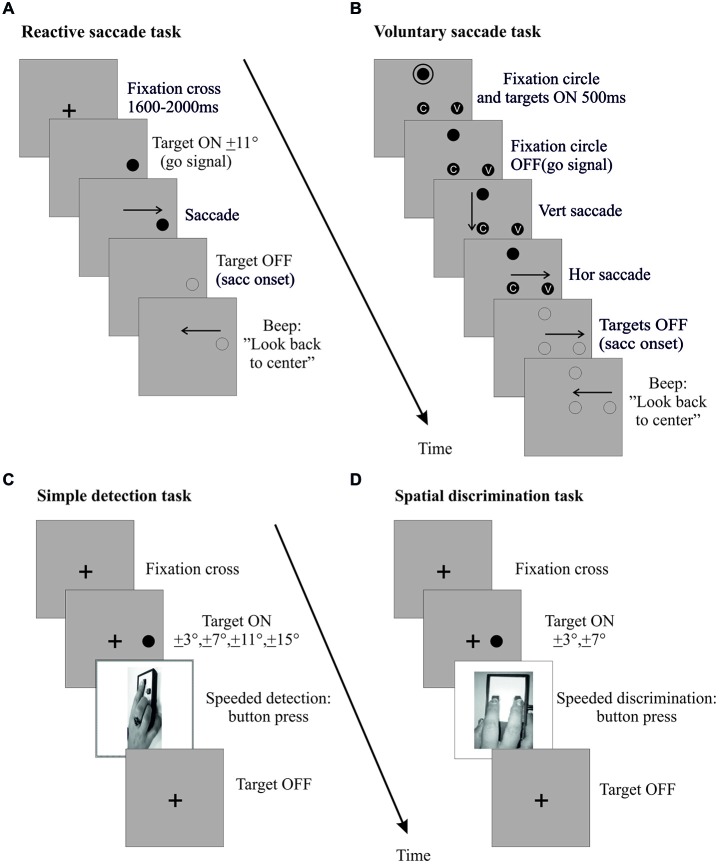
**Pre- and post-exposure phases**. Sequences of events in right hemi-field trials (left hemi-field trials not shown) are plotted for the reactive saccades task **(A)**, the voluntary saccades task **(B)**, the simple detection task **(C)** and the spatial discrimination task **(D)**. For the saccade tasks **(A,B)**, the procedure was the same as in the corresponding exposure phase, except that the lateral target was randomly presented at 7° or 11° and was turned off at saccade onset. In the simple detection task **(C)**, subjects were instructed to press a button with their right hand as soon as they detected a peripheral visual target, while keeping their eyes on a central fixation cross throughout the trial. The target was presented at a random position along the horizontal meridian, in the left or right hemi-field (3°, 7°, 11° or 15°). The target disappeared when subjects pressed the response button. In the spatial discrimination task **(D)**, subjects also provided a right hand response as soon as they detected a peripheral visual target while maintaining central fixation, but were further instructed to select the left key or the right key when the target was presented in the left or right hemi-field (3° or 7°), respectively. The target disappeared when subjects pressed the response button.

Each pre- and post-exposure phase comprised a saccadic task (Figures 3A,B) and a simple detection task (Figure 3C). In the saccadic task, subjects performed 24 saccade trials (12 rightward and 12 leftward, randomly interleaved). The design of this saccadic task was similar to that of the corresponding (reactive or voluntary) exposure phase, except that once the horizontal saccade was detected (eye velocity threshold: 70–90°/s), the visual display was turned off to suppress any visual feedback, and a beep occurring 500 ms later informed subjects to look back to the center.

The simple detection task, designed to measure exogenous shifts of covert attention, involved 3 blocks of 48 trials. As shown in Figure 3C, subjects were instructed to fixate a cross at the center of the screen. After 800, 1600 or 2000 ms, a peripheral target appeared randomly at one of eight different locations (3°, 7°, 11°, 15°, either left or right) along the horizontal meridian. Participants had to maintain their gaze on the FC throughout the trial and to respond by pressing a button with their dominant hand as soon as they detected the target, which disappeared at button-press (or after a fixed delay of 500 ms in case of no response). Eye position was monitored online and a warning beep sounded whenever subjects moved their eyes more than 1° away from the FC, in which case the trial was excluded from analysis.

#### Data Analysis

Eye movement data were analyzed off-line using custom software developed in Matlab v.7.1 (Math Works Inc., Natick, MA, USA). Data from the left and right eyes were averaged. The start and end positions of each primary horizontal saccade were identified based on a velocity threshold of 50°/s, and were used to compute saccadic amplitude and gain. Saccadic amplitude was the difference between the initial and the final positions of the eye. Saccadic gain was calculated by dividing saccade amplitude by target retinal eccentricity (difference between the target position and the starting position of the saccade). The mean saccadic gain was obtained separately for each group, for rightward and leftward saccades and for pre- and post-exposure phases. Then, the gain change between pre- and post-exposure was calculated separately for the adapted and non-adapted hemi-fields, and for the reactive and voluntary saccades. A negative value indicates an increase—whereas a positive value indicates a decrease—of saccadic gain in the post-phase relative to the pre-exposure phase. As a significant gain change was found in the adaptation tasks of each individual participant, all subjects were included for subsequent analyses. Trials with saccades that were not correctly detected or were contaminated with blink were eliminated, as well as trials with a saccade gain outside the range of mean ± 3 SD. Eliminated trials represented 3% (SEM ± 0.29) of the total number of RS trials and 5.57% (SEM ± 0.29) of the total number of VS trials.

In the simple detection task, manual reaction time (RT) was defined as the time elapsed between the target onset and the button press. The median RT was calculated separately for each subject, for the four target eccentricities (3°, 7°, 11° and 15°), the two hemi-fields (left and right) and for the pre- and post-exposure phases, and then averaged in each group of subjects. An attention deployment index was calculated as the difference of manual RT between pre- and post-exposure phases and expressed as a percentage relative to the pre-exposure value. Thus, a negative value indicates an increase (i.e., slower response), whereas a positive value indicates a decrease (i.e., faster response) of manual RT in the post- relative to the pre-exposure phase. This polarity of calculation was chosen such that the index logically represents variations of performance, and we applied the same convention for the calculation of gain changes to ease the comparison of these oculomotor and detection performance changes (see Figure 7).Trials where subjects failed to maintain eye position within 1° of the FC or responded faster than 200 ms or slower than 500 ms were eliminated (representing 7%, SEM ± 1.27 and 5.98%, SEM ± 0.71 of the total number of detection trials in RS and in VS conditions, respectively).

Statistical analyses were performed with Statistica 9 (Statsoft Inc., Tulsa, OK, USA). First, to quantify saccadic adaptation, repeated-measures ANOVAs were performed on the mean saccadic gain measured separately in each group of subjects, with the factors Phase (pre, post) and Hemi-field (exposed, unexposed). Similarly, for the simple detection task, the median manual RT was submitted to repeated-measures ANOVAs separately for each group, with the factors: Phase (pre, post), Hemi-field (exposed, unexposed) and Target eccentricity (3°, 7°, 11°, 15°). Finally, the attention deployment index was submitted to an ANOVA testing the factors Type of exposure (adaptation, control), Hemi-field (exposed, unexposed) and Target eccentricity (3°, 7°, 11°, 15°). *Post hoc* Fisher’s least significant difference (*LSD*) tests were used to explore significant interactions. Significance was set at *p* < 0.05. Values are reported as mean ± 1 SEM. All data from the saccadic tasks and simple detection task were normally distributed, as verified by Lilliefors test, and the homogeneity of variances was confirmed using the Hartley test.

### Experiment II

The objectives of Experiment II were: (1) to provide internal replication of the novel effect of oculomotor plasticity on visual detection performance discovered in Experiment I (see “Results” Section); (2) to confirm the attentional nature of this effect by testing its generalization to a spatial discrimination task; and (3) to provide definitive and robust evidence for the novel finding by adopting a strictly controlled within-subject design.

#### Subjects

Fourteen healthy volunteers (9 females and 5 males, all right-handed, mean age: 26.4, SEM ± 0.5) performed two sessions of leftward RS (adaptation session and control session) in a counterbalanced order, separated by 1–2 weeks.

#### Apparatus and Procedure

The apparatus and procedure were similar to those in Experiment I, with only a few differences detailed hereafter. To adhere to a within-subject design, participants underwent both the critical experimental manipulation (adaptation of RS in the left hemi-field) and the control task (execution of RS in the left hemi-field). In addition, we used a speeded spatial discrimination task as a proxy for the deployment of exogenous attention during the pre-exposure and post-exposure phases (Figure 3D). In this task, a peripheral target appeared randomly at one of four different locations (3°, 7°, either left or right), and participants had to indicate in which hemi-field (left or right) the target appeared by a speeded left or right key-press, respectively. They performed the task with the middle and index finger of their dominant hand acting on a two-button response box, located along their body midline, while maintaining their gaze on the central FC. The task consisted of four blocks of 48 trials (Figure 1).

#### Data Analysis

Five subjects in whom the gain of leftward or rightward saccades varied significantly in the control session were excluded from further analyses. The same exclusion criteria as in Experiment I led to the rejection of 8% (SEM ± 0.88) of saccade trials in the adaptation session and of 8% (SEM ± 0.82) in the control session and to 9% (SEM ± 1.26) and 9.17% (SEM ± 1.03), respectively, of discrimination trials (including 1.57% SEM ± 0.27 and 1.39% SEM ± 0.24 due to wrong answer).

Statistical analyses of Experiment II relied on a full within-subject design. To quantify saccadic adaptation, repeated-measures ANOVAs were performed on the mean saccadic gain with the following three within-subject factors: Session (adaptation, control), Hemi-field (left, right) and Phase (pre, post). For the spatial discrimination task, the median manual RT was submitted to a four-way repeated measures ANOVAs [same as for the saccadic gain, with the additional within-subject factor: Target eccentricity (3°, 7°)]. The same attention deployment index as in Experiment I was calculated and submitted to an ANOVA with three within-subject factors: Session (adaptation, control), Hemi-field (left, right) and Target eccentricity (3°, 7°).

## Results

### Experiment I

#### Oculomotor Performance

**Figure 4:**
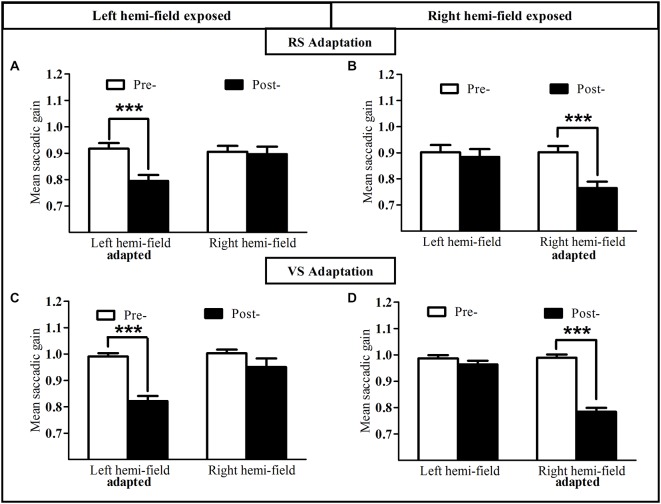
**Oculomotor performance in the reactive and voluntary saccade conditions of Experiment I**. Mean saccadic gain are shown in the pre- (white bars) and post-exposure phases (black bars). Upper row: reactive adaptation task of the Adapt-Left group **(A)** and Adapt-Right group **(B)**; lower row: voluntary adaptation task of the Adapt-Left group **(C)** and the Adapt-Right group **(D)**. Error bars indicate ±1 SEM. Asterisks denote significant differences between pre- and post- phases (*Post hoc* LSD test, *p* < 0.001).

The mean gain of saccades measured in the pre-exposure and post-exposure phases was analyzed separately for RS (Figures 4A,B) and VS (Figures 4C,D). As shown in Figures 4A,B, the gain of RS directed toward the exposed hemi-field was reduced after the adaptation exposure. Indeed, a two-way repeated-measures ANOVA with the factors Phase (pre- vs. post-) and Hemi-field (left vs. right) revealed a significant interaction between these two factors, both for the Adapt-Left group [*F*_(1,9)_ = 46.11, *p* = 0.00008; Figure 4A] and the Adapt-Right group [*F*_(1,9)_ = 54.20, *p* = 0.00004; Figure 4B]. *Post hoc* LSD tests indicated a significant decrease of saccadic gain in the post- relative to the pre-phase in the exposed hemi-field for both groups (0.79 ± 0.02 vs. 0.91 ± 0.02 and 0.76 ± 0.02 vs. 0.90 ± 0.02, respectively, both *p* < 0.0001). In contrast, no significant difference was found in the unexposed hemi-field in either group [respectively *p* = 0.47, and *p* = 0.15]. In addition, saccadic gain changes in the exposed hemi-field did not differ between the Adapt-Left and Adapt-Right groups (unpaired *t*-test, *p* = 0.34). Finally, concerning the saccade control groups, the same two-way ANOVA disclosed no significant main effect of Phase or Hemi-field, nor any interaction between these factors [Control-Left group *p* = 0.18 and Control-Right group *p* = 0.73].

This pattern of results was replicated for the VS condition (Figures 4C,D). Indeed the two-way repeated-measures ANOVA revealed a significant interaction between the Phase factor (pre- vs. post-) and the Hemi-field factor (left vs. right), both for the Adapt-Left group [*F*_(1,9)_ = 7.49, *p* = 0.023; Figure 4C] and the Adapt-Right group [*F*_(1,9)_ = 136.66, *p* < 0.00001; Figure 4D]. *Post hoc* LSD tests indicated a significant decrease of saccadic gain in the post- relative to the pre-phase in the exposed hemi-field for both groups (0.82 ± 0.02 vs. 0.99 ± 0.01 and 0.78 ± 0.01 vs. 0.99 ± 0.01, respectively, both *p* < 0.0001). In contrast, no significant difference was found in the unexposed hemi-field in either group [respectively *p* = 0.12, and *p* = 0.058]. In addition, saccadic gain changes in the exposed hemi-field did not differ between the Adapt-Left and Adapt-Right groups (unpaired *t*-test, *p* = 0.61). Concerning the saccade control groups, the same two-way ANOVA disclosed no significant main effect of Phase or Hemi-field, nor any interaction between these factors [Control-Left group *p* = 0.48 and Control-Right group *p* = 0.21].

In conclusion, for both reactive and VS conditions, the gain of saccades in the exposed hemi-field –but not in the unexposed hemi-field- was significantly reduced after the adaptation phase, whereas no such gain change was observed in the control task in either the exposed or the unexposed hemi-field.

#### Detection Performance

**Figure 5:**
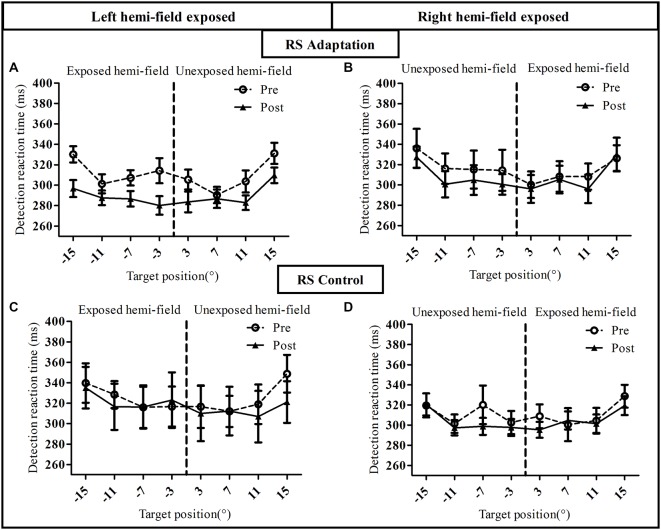
**Visual detection performance in the reactive saccade condition of Experiment I**. Mean manual reaction time is plotted as a function of target position for the pre-exposure phase (open circles, dotted line) and post-exposure phase (filled triangles, solid line). Upper row: adaptation groups (**A**: Adapt-Left, **B**: Adapt-Right group), lower row: control groups (**C**: Control-left group, **D**: Control-right group).

**Figure 6:**
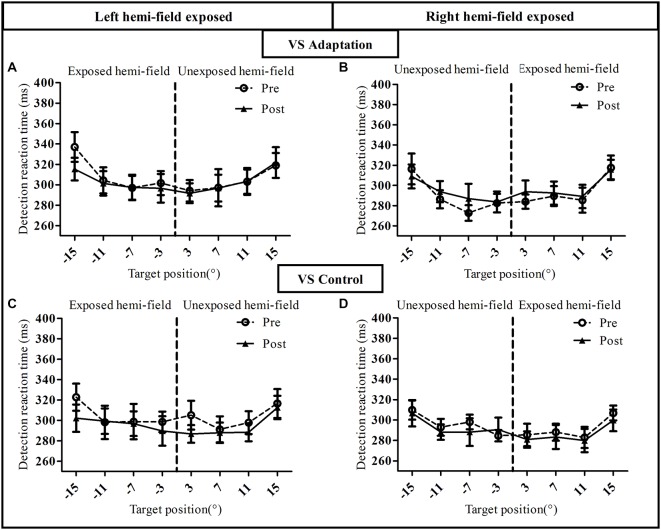
**Visual detection performance in the voluntary saccade condition of Experiment I**. Mean manual reaction time is plotted as a function of target position for the pre-exposure phase (open circles, dotted line) and post-exposure phase (filled triangles, solid line). Upper row: adaptation groups (**A**: Adapt-Left group, **B**: Adapt-Right group), lower row: control groups (**C**: Control-left group, **D**: Control-right group).

To assess the influence of saccadic adaptation on covert shifts of attention, we analyzed, separately for the RS condition (Figure 5) and the VS condition (Figure 6), the manual RT in the speeded simple detection task performed before and after the exposure phase. As shown in Figure 5, a three-way ANOVA with the factors Phase, Hemi-field and Target eccentricity revealed a significant effect of Target eccentricity in all groups (Figure 5A): [*F*_(3,27)_ = 16.51, *p* < 0.00001], (Figure 5B): [*F*_(3,27)_ = 18.90, *p* < 0.00001], (Figure 5C): [*F*_(3,27)_ = 8.17, *p* = 0.0005 and (Figure 5D): *F*_(3,27)_ = 9.50, *p* = 0.0002], due to longer RTs for the most eccentric target (±15°) relative to other targets. In addition, a significant effect of phase [*F*_(1,9)_ = 12.29, *p* = 0.007] and a significant interaction between Hemi-field and Phase [*F*_(1,9)_ = 5.61, *p* = 0.042] was revealed only for the Adapt-Left group. This interaction is due to a larger decrease in RT between post- and pre- phases in the left hemi-field as compared to the right hemi-field. In contrast, there was no significant effect of Phase in any of the other three groups [Adapt-right group: (*F*_(1,9)_ = 1.60, *p* = 0.24), Control-left group: (*F*_(1,9)_ = 0.65, *p* = 0.44) and Control-right group: (*F*_(1,9)_ = 0.28, *p* = 0.60)]. Finally, for the Control-right group, the interaction between the three factors was significant [*F*_(3,27)_ = 4.07, *p* = 0.016] and related to pre- vs. post-exposure differences at 7° in the left hemi-field and at 3° in the right hemi-field.

This pattern of results was replicated for the VS condition except one major difference (Figure 6). Indeed, the three-way ANOVA revealed a significant effect of Target eccentricity in all groups [Adapt-Left group: (*F*_(3,27)_ = 23.05, *p* < 0.00001), Adapt-Right group: (*F*_(3,27)_ = 18.58, *p* < 0.00001), Control-Left group: (*F*_(3,27)_ = 9.91, *p* = 0.0001) and Control-Right group: (*F*_(3,27)_ = 11.79, *p* = 0.00004)], an effect due to longer RTs for the 15° target as compared to the other targets. However, contrary to the RS condition (Figure 5), the effect of Phase was not significant in any of the four groups (all *p* > 0.3) as the pre- and post-exposure values overlapped almost perfectly. Importantly, the mean RT at baseline (pre-adaptation phase) was consistent across all 8 groups, as the three-way ANOVA with the between subjects factor Group and the two within subject factors Hemi-field (exposed, unexposed) and Target eccentricity (3°, 7°, 11°, 15°), showed no significant effect of Group [*F*_(7,72)_ = 0.75, *p* = 0.63].

**Figure 7:**
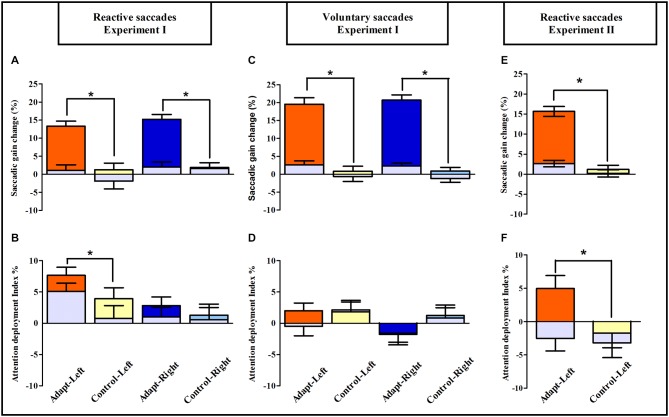
**Saccadic adaptation boosts pure covert attention: summary of oculomotor and visuo-attentional performance in Experiments I and II**. Upper row: saccadic gain changes between pre- and post-exposure are plotted separately for the Adapt-Left group (orange), Control-Left group (yellow), Adapt-Right group (blue) and Control-Right group (light blue) in the reactive **(A)** and voluntary conditions **(C)** of Experiment I and in the reactive condition of Experiment II **(E)**; gray bars represent the unexposed hemi-field in all groups. Asterisks indicate significant differences between the adaptation and control groups for the exposed hemi-fields (*post hoc* LSD tests, all *p* < 0.0001). Error bars indicate ±1 SEM. Lower row: the mean attention deployment index averaged across target positions is plotted separately for the Adapt-Left group (orange), Control-Left group (yellow), Adapt-Right group (blue) and Control-Right group (light blue) in the reactive **(B)** and the voluntary conditions **(D)** of Experiment I and in the reactive condition of Experiment II **(F)**; gray bars represent the unexposed hemi-field. Asterisks denote significant differences between adaptation and control groups (*post hoc* LSD tests, all *p* < 0.05). Error bars indicate ±1 SEM.

To further quantify the net effect of adaptation on the simple detection task, the relative change in manual RTs between pre- and post-exposure was used to compute an attention deployment index (see “Materials and Methods” Section). As shown in Figures 7B–D, the pattern of the attention deployment index differed from the pattern of oculomotor changes reported above and re-plotted in Figures 7A–C. Compared to controls, the attention deployment index was indeed higher, thus indicating faster simple detection, only after adaptation of leftward RS for stimuli presented in the left hemi-field (Figure 7B). This observation was substantiated by an ANOVA with the factors Exposure (adapt, control), Hemi-field (exposed, unexposed) and Target eccentricity (3°, 7°, 11°, 15°) which revealed only one significant effect concerning the Exposure × Hemi-field interaction [*F*_(1,18)_ = 6.59, *p* = 0.019]. Since the same three-way ANOVA revealed no significant source of variance in any of the other groups, these findings imply a specific boosting of leftward covert attention by adaptation of leftward RS. Notably, this improvement came at no cost for the opposite (non-adapted) right hemi-field, where subjects maintained their capability of covertly deploying attention. This highly specific pattern also rules out any effect on manual RTs of practice or fatigue, as these effects would have uniformly affected performance in all groups and both hemi-fields. Rather these results provide evidence for the role of RS adaptation in the deployment of spatial attention in a simple detection task.

### Experiment II

#### Oculomotor Performance

**Figure 8:**
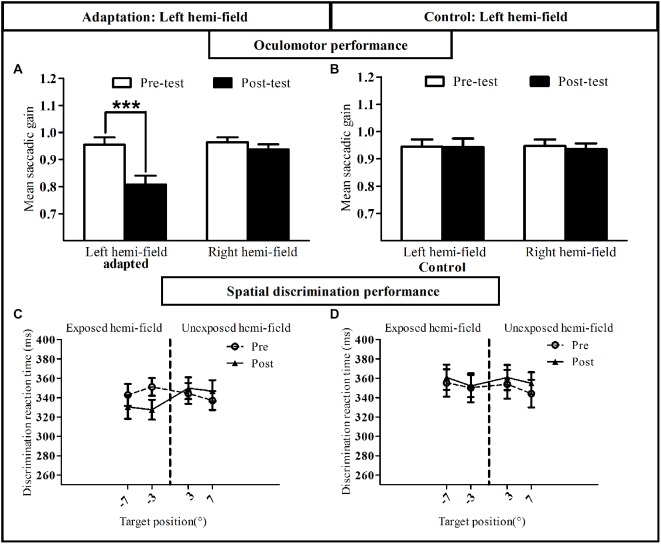
**Oculomotor and visual discrimination performance in Experiment II**. Upper row: mean saccadic gain is shown in the pre-(white bars) and post-exposure phases (black bars) in the reactive saccade adaptation session **(A)** and in the control session **(B)**. Lower row: mean manual reaction time is plotted as a function of target position for the pre-exposure phase (open circles, dotted line) and post-exposure phase (filled triangles, solid line) in the reactive saccade adaptation session **(C)** and in the control session **(D)**. Error bars indicate ±1 SEM. Asterisks denote significant difference between pre- and post- phases (*Post hoc* LSD test, *p* < 0.001).

To assess oculomotor performance in adaptation and control sessions (Figures 8A,B) the mean gain of RS measured in the pre-exposure and post-exposure phases was submitted to a three-way repeated-measures ANOVA with the factors Session (adaptation, control), Phase (pre-, post-) and Hemi-field (left, right). A significant interaction between these three factors was found [*F*_(1,8)_ = 123.18, *p* < 0.00001]. *Post hoc* LSD tests indicated a significant decrease of saccadic gain in the post- relative to the pre-phase in the left exposed hemi-field for the adaptation session (0.95 ± 0.03 vs. 0.80 ± 0.03, *p* = 0.0001). In contrast, no significant difference was found in the non-adapted hemi-field and as expected, in both hemi-fields in the control session (all* p* > 0.07). Thus, as in Experiment I, participants were significantly adapted for leftward RS, without transfer to (rightward) saccades to the non-adapted hemi-field.

#### Spatial Discrimination Performance

Average manual RT recorded in the speeded spatial discrimination task were submitted to a four-way ANOVA with Session, Phase, Hemi-field and Target eccentricity (3°, 7°) as within-subject factors. As shown in Figures 8C,D, this analysis disclosed a significant interaction between the factors Session, Phase and Hemi-field [*F*_(1,8)_ = 5.58, *p* = 0.046], with no significant four-way interaction [*F*_(1,8)_ = 0.29, *p* = 0.60]. *Post hoc* LSD tests indicated a significant decrease of RT in the post-exposure relative to the pre-exposure phase in the left hemi-field for the adaptation session (*p* = 0.01). In addition, a three-way ANOVA on the attention deployment index (see “Materials and Methods” Section) with the factors Session (adaptation, control), Hemi-field (left, right) and Target eccentricity (3°, 7°) revealed only one significant effect concerning the Session × Hemi-field interaction [*F*_(1,8)_ = 5.80, *p* = 0.043]. In keeping with the results of Experiment I, this interaction was due to the attention deployment index being higher, relative to control, only for stimuli presented in the left hemi-field and only after the RS adaptation session (*Post hoc* LSD, *p* = 0.005; Figure 7F). The session- and hemi-field-specific pattern again rules out effects on RTs potentially due to practice or fatigue, and demonstrates that adaptation of RS can also improve visual performance in a spatial discrimination task.

## Discussion

This study aimed at establishing whether saccadic adaptation, by changing the metrics of eye movements, also changes the covert orienting of exogenous visuospatial attention. Participants’ speeded detection and discrimination performance was measured in tasks summoning covert exogenous attention shifts toward unpredictable visual stimuli while continuously maintaining central ocular fixation. As previously reported (Abel et al., 1978; Miller et al., 1981; Deubel et al., 1986; Albano, 1996; Hopp and Fuchs, 2004; Alahyane et al., 2007; Pélisson et al., 2010; Prsa and Thier, 2011; Herman et al., 2013), saccadic adaptation elicited by the double-step paradigm was directionally-specific, as all four adaptation groups in Experiment I and the adaptation session in Experiment II showed a decrease in saccadic gain in the adapted hemi-field, with no transfer to saccades in the opposite hemi-field (Figures 7A,C,E). The amount of adaptation was slightly larger for VS (20.1%) than for RS (14.2% in Experiment I and 15.6% in Experiment II), but comparable across adapted hemi-fields, and no significant gain change in either hemi-field was observed after mere execution of saccades in any of the four control groups in Experiment I. Crucially, the results from both the speeded detection and spatial discrimination tasks of Experiments I and II show that after adaptation of leftward RS the performance index increased relative to the control task, specifically in the adapted hemi-field. These replicated findings provide robust evidence for the role of leftward RS adaptation in the deployment of covert spatial attention.

One possible interpretation of this newly-demonstrated functional link between RS adaptation and covert attention shifts is that the internal representation of targets presented during the detection task had been shifted by saccadic adaptation. Given the positive scaling of detection RT with target eccentricity, an inward shift (reduced eccentricity) of the target internal representation following backward adaptation could in theory yield better performance (reduced detection RT reflecting an “horizontal shift” of the curve). Note however that this theoretical explanation is hardly compatible with our data. First, the relationship between detection RT and target eccentricity (see Figures 5A, 8C) does not exhibit a scaling of RT over eccentricity sufficient to explain the observed reduction of RT solely by an “horizontal shift” of the curve, as the effect of target eccentricity is seen starting only between 11° and 15° in Figure 5A, and is virtually absent in Figure 8C. Rather the curves depicted in Figures 5, 8 seemed to shift vertically (downward) after adaptation. Second, we found no correlation in our sample of subjects between saccade adaptation magnitude and attention performance (Experiment I: *R* = 0.02; *p* = 0.95 and Experiment II: *R* = 0.52, *p* = 0.15). Third, an adaptation-related shift of targets internal representation is more likely to take place after adaptation of VS rather than of RS because effects of adaptation on visual perception have been preferentially demonstrated in the former case, as will be discussed in the next section.

Compared to previously reported effects of saccade plasticity on pre-saccadic shifts of attention (Ditterich et al., 2000; Doré-mazars and Collins, 2005; Collins and Doré-Mazars, 2006; Collins et al., 2010; Khan et al., 2010), the present findings clearly point to an oculomotor-attentional phenomenon which differs from previously reported effects of saccadic plasticity on pre-saccadic shifts of attention by the following aspects. First, it is un-related to any execution of saccades or even to any oculomotor preparation, as subjects kept central fixation throughout the simple detection and spatial discrimination tasks. Second, it is not limited to the location corresponding to the adapted saccade endpoint, visuospatial attention being boosted irrespective of target eccentricity (Figures 5A, 8C). This finding is consistent with the broad size of adaptation fields elicited by adaptation of a single saccade vector (Frens and van Opstal, 1994; Noto et al., 1999; Collins et al., 2007; Alahyane et al., 2008), which in our paradigm could encompass all targets surrounding the 11° adapted position and used in our simple detection and spatial discrimination tasks. Note however that changes in the spatial distribution of attention do not necessarily follow the adaptation field, indeed the decrease in RT across the hemi-field is rather uniform as compared to the spatial distribution of saccade kinematic changes (adaptation field). Third, while changes of pre-saccadic shifts of attention following adaptation were previously illustrated both when saccades were triggered in a reactive mode (Collins and Doré-Mazars, 2006; Collins et al., 2010; Khan et al., 2010) and in a voluntary mode (Doré-mazars and Collins, 2005; Collins and Doré-Mazars, 2006), here we demonstrate that the deployment of covert shifts of attention is modified only after adaptation of RS. Though beyond the scope of the present study, it would be interesting to determine whether VS adaptation can affect endogenous visual attention. This transfer is predicted by the neurophysiological interpretation discussed in the next section. Another argument for such transfer is that the visual stimulation used to elicit endogenous attention is necessarily less transient (for example subjects may have to report a modification of an intrinsic visual feature—e.g., shape or contrast—of a target presented a few hundreds of ms ealier), than the brisk target presentation used to measure exogenous attention in the present study. In this respect, Zimmermann and Lappe (2009) proposed that the amount of adaptation transfer from reactive and voluntary saccades to a visual localization task is larger when targets used during the localization and adaptation tasks are more similar. In sum, we provide the first evidence for a specific functional link between RS adaptation and pure covert attention shifts. Note that this coupling could in theory also be detected in the opposite direction, as an effect of attention on saccadic adaptation. This possibility was first raised by McFadden et al. (2002) who showed that covert attention shifts elicited exogenously in a cued discrimination task can be modified by an adaptation-like procedure and that such adaptation of attention transfers to saccades. We also provided recent evidence supporting this hypothesis by demonstrating that attention mobilization by a discrimination task performed simultaneously with the adaptation protocol has a beneficial effect on the level of adaptation (Gerardin et al., under revision).

Since both tasks required covert exogenous attention (detection/discrimination of an un-cued peripheral visual target), our findings concur in supporting the recent proposal that the premotor theory of attention holds for exogenous attention, but not endogenous attention (Smith and Schenk, 2012). Our results also substantiate the recent claim that neural changes related to saccadic adaptation can spill over visual perception, as inferred from errors observed in tasks involving visual localization (Zimmermann and Lappe, 2009), visually-guided hand pointing movements (Cotti et al., 2007; Hernandez et al., 2008) and generation of anti-saccades elicited by targets in the adapted hemi-field (Cotti et al., 2009, but see Collins et al., 2008). However, with the exception of one study by Zimmermann and Lappe (2010) who used a RS adaptation protocol different from the classical double-step protocol used here, these errors were predominantly manifest following adaptation of VS (Cotti et al., 2007; Zimmermann and Lappe, 2009), further corroborating the distinctive nature of the attentional improvement reported here.

The direction-specific effect of saccade adaptation on covert exogenous attention (Figure 7) is reminiscent of the asymmetrical cognitive effect produced by adaptation of manual pointing movements to prism-induced optical deviation (Rossetti et al., 1998): while adaptation to leftward-deviating prisms induces neglect-like rightward biases in visuospatial tasks in healthy individuals (Colent et al., 2000; Loftus et al., 2009; Bultitude and Rafal, 2010; Bultitude et al., 2013), right-deviating prisms do not induce leftward visuospatial biases (Michel, 2006; Bultitude et al., 2013; Schintu et al., 2014). Both prismatic and saccadic adaptations exert direction-selective effects on visuo-spatial perception that may depend upon attentional specialization of the right cerebral hemisphere. Thus, in complement to prismatic adaptation, saccadic adaptation may offer new solutions for rehabilitation of spatial attention deficits in unilateral neglect. In addition, based on these observations, the results of the present work can be framed within a model in which the known specialization of the right hemisphere for orienting spatial attention relies on two possibly dissociable systems: a system orienting attention to the right, which is spared in neglect, although exerting an abnormally strong attention due to pathological hemispheric imbalance (Snow and Mattingley, 2006), and a system orienting attention to the left, which is damaged in neglect (Corbetta and Shulman, 2002; Charras et al., 2012). On the basis of the present findings, we suggest that the latter attention system can be boosted by RS adaptation, possibly due to shared neural substrates. Indeed, exogenous attention involves regions in the dorsal and ventral attention networks, which are known to be mainly lateralised in the right hemisphere (Nobre et al., 2000; Corbetta and Shulman, 2002; Husain and Nachev, 2007; Corbetta et al., 2008; Thiebaut de Schotten et al., 2011; Bartolomeo et al., 2012). In addition, adaptation of leftward RS activates structures of the contralateral right hemisphere (Gerardin et al., 2012) that overlap, at the level of the Temporo-Parietal Junction (TPJ), the ventral attentional network responsible for reorienting of exogenous covert attention following presentation of behaviorally relevant stimuli (Nobre et al., 2000; Corbetta et al., 2008). Most notably, adaptation of VS does not elicit any significant activity in the same right TPJ region (Gerardin et al., 2012), in agreement with the absence of attentional effects found here following VS adaptation. We therefore suggest that the relative functional specialization/lateralization of TPJ may be responsible for the specificity of the attentional effects reported in the present study. The ineffectiveness of VS adaptation in boosting covert exogenous attention is also consistent with VS adaptation-dependent recruitment of a more dorsal parietal network, centred over the intra-parietal sulcus (Gerardin et al., 2012; Panouillères et al., 2014), which has been previously involved in endogenous attention [Lateral Intra-Parietal area in monkey (Wardak et al., 2004; Balan and Gottlieb, 2009); Intra-Parietal Sulcus in humans (Jerde et al., 2012)]. In summary, this model provides a parsimonious framework for linking these behavioral findings to their possible neural underpinnings, which by no means excludes the contribution of other brain areas involved both in covert attention [e.g., Frontal Eye Fields (Moore and Fallah, 2001); Superior Colliculus (Zénon and Krauzlis, 2012; Katyal and Ress, 2014); cerebellar vermis (Baier et al., 2010)] and saccadic adaptation (see Hopp and Fuchs, 2004; Iwamoto and Kaku, 2010; Jenkinson and Miall, 2010; Pélisson et al., 2010; Prsa and Thier, 2011).

In conclusion, this work provides previously unavailable evidence that the functional relationships between saccadic and covert attention systems go beyond the spatially-limited visual enhancement occurring just before saccadic execution.

## Author Contributions

Conceived and designed the experiments: OH, AF and DP. Performed the experiments: OH, ER and RM. Analyzed the data: OH, ER and RM. Wrote the paper: OH, AF and DP.

## Conflict of Interest Statement

The authors declare that the research was conducted in the absence of any commercial or financial relationships that could be construed as a potential conflict of interest.
